# A DFT investigation of the blue bottle experiment: *E*^∘^_half-cell_ analysis of autoxidation catalysed by redox indicators

**DOI:** 10.1098/rsos.170708

**Published:** 2017-11-08

**Authors:** Taweetham Limpanuparb, Pakpong Roongruangsree, Cherprang Areekul

**Affiliations:** 1 Science Division, Mahidol University International College, Mahidol University, Salaya, Nakhon Pathom 73170, Thailand; 2 National Nanotechnology Center (NANOTEC), National Science and Technology Development Agency, Khlong Luang, Pathum Thani 12120, Thailand; 3 Department of Chemical Engineering, McGill University, Quebec H3A 0C5, Canada

**Keywords:** blue bottle experiment, reduction potential, density functional theory, chemical pattern formation, reaction mechanism

## Abstract

The blue bottle experiment is a collective term for autoxidation reactions catalysed by redox indicators. The reactions are characterized by their repeatable cycle of colour changes when shaken/left to stand and intricate chemical pattern formation. The blue bottle experiment is studied based on calculated solution-phase half-cell reduction potential of related reactions. Our investigation confirms that the reaction in various versions of the blue bottle experiment published to date is mainly the oxidation of an acyloin to a 1,2-dicarbonyl structure. In the light of the calculations, we also propose new non-acyloin reducing agents for the experiment. These results can help guide future experimental studies on the blue bottle experiment.

## 1. Introduction

**Figure 1.**
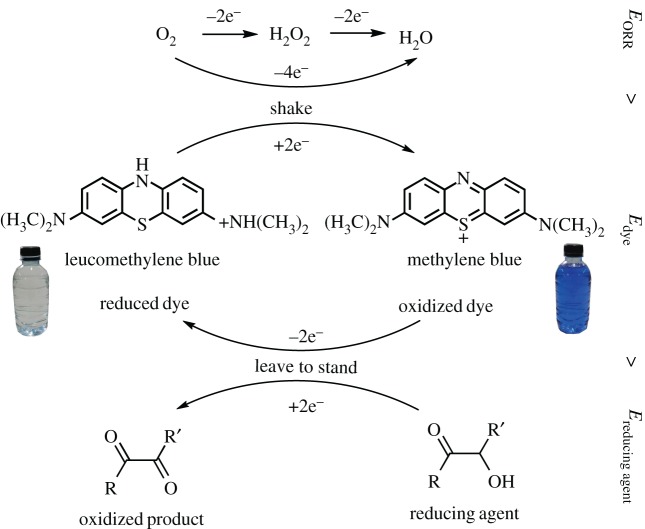
The general framework of the blue bottle experiment.

**Figure 2.**
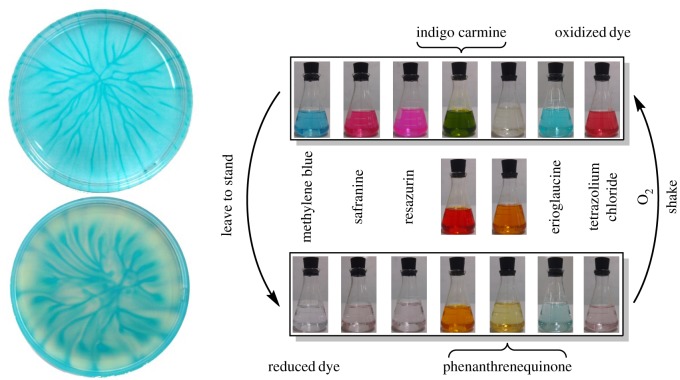
Patterns formed in a green version of the reaction (ascorbic acid is reducing agent) and colours of various dyes in the rapid version of the reaction (benzoin is reducing agent). Adapted with permission from Rajchakit and Limpanuparb [7,8]. Copyright © 2016 American Chemical Society.

Autoxidation of reducing agents catalysed by redox indicators have been reported in the literature since 1946 [1,2]. The most notable reaction is the ‘blue bottle experiment’ [3], an oxidation of glucose catalysed by methylene blue under an alkaline condition [4]. For bulk reaction in a flask, methylene blue is reduced to colourless (leuco) form by the aldose sugar when left to stand and is oxidized to blue form by atmospheric oxygen when shaken. The cycle can be repeated many times before the reactants run out or the solution turns brown due to side reactions [5]. For thin-layer reaction in a Petri dish, dot and line patterns of the oxidized indicator develop over a period of time [6–8]. Figure 1 shows the general reaction framework and figure 2 shows patterns and colours observed in different variations of the reaction.

The blue bottle reaction and its analogues are popular chemical demonstrations due to their visual appeal and simplicity, and the majority of the research on this topic is published in the *Journal of Chemical Education* [1–18]. Fundamental research and non-education applications of the reactions have also been discussed elsewhere [19–26].

In essence, there were attempts to test new reducing agents [7,11,16] and indicators [7,8], to elucidate the mechanism and kinetics of the reactions [5,11] and to model the pattern formation [6,19–23,26]. Pattern formation may be comparable to the Belousov–Zhabotinsky [27] and the Briggs–Rauscher [28] reactions. However, the blue bottle model is relatively simple and versatile because it requires only a few reactants and many alternative reagents can produce similar results.

Despite numerous reports, the understanding of the reaction is advanced incrementally by mostly trial-and-error experiments. A number of papers reported only one new reducing agent [16] or indicator [9,10,17] or pattern formation in one specific system [6]. The reports can also be conflicting or incomplete. For example, in 2012, Anderson *et al*. [5] suggested that gluconate is not a major product and the reaction may produce hydrogen peroxide, but later work [26] as late as 2014 still discusses the gluconate compound as the main product; in 1974, Chen [29] reported the use of indophenol as a dye for the experiment, but, in 2016, Rajchakit & Limpanuparb [7] failed to reproduce it. Experimental reports usually mention only a structure of a dye in its solid form but do not explicitly show oxidized or reduced form(s) of the compound [7,17] and there was no experimental identification of the products in all cases except one [5].

In this first density functional theory (DFT) investigation of the blue bottle experiment, we aim to propose a theoretical framework to resolve discrepancies in the current literature and guide future experimental studies. The manuscript is structured as follows: Methodology describes reactions and computation approach; Results and discussion presents the main results based on reduction potentials, and preliminary experimental evidence, detailed computational/experimental results are given as the electronic supplementary material; Concluding remarks and future work are discussed at the end of the paper.

## 2. Methodology

### 2.1. Preliminary consideration

Figure 1 shows that there are three main groups of reactions in the blue bottle experiment: oxygen reduction reactions (ORRs), oxidation/reduction of redox indicators and oxidation of reducing agents. It is natural to characterize these redox reactions in terms of standard half-cell potential, $E_{\mathrm{half}\text{-}\mathrm{cell}}^{\circ }$ in aqueous solution at 298.15 K. Because $E_{\mathrm{half}\text{-}\mathrm{cell}}^{\circ }$ is a ‘per electron’ quantity, it conveniently allows quick comparison and helps with our prediction whether a compound can possibly be used in a blue bottle reaction. By considering the potentials, it is equivalent to the consideration of Gibbs energy. A reaction is spontaneous provided that the cell potential made by combination of reduction potentials of two half-reactions,

2.1 $$E_{\mathrm{cell}}=E_{\mathrm{cathode}}-E_{\mathrm{anode}}$$

is positive. In other words*,* a necessary but not sufficient condition for the combination of ORR, oxidation/reduction of dyes and oxidation of reducing agents to make up a blue bottle experiment is

2.2 $$E_{\mathrm{ORR}}>E_{\mathrm{dye}}>E_{\mathrm{reducing}\;\mathrm{agent}}$$

as shown in figure 1. All the discussions that follow use the same potential comparison process as a thinking framework.

We include representative compounds reported in the blue bottle literature and possible reagents to explore alternative redox indicators/reducing agents and to gain mechanistic insight of the reaction. Table 1 lists the oxidized and reduced structures of all compounds in this study. If oxidized/reduced form(s) of the compounds are not explicitly mentioned in the literature, we do our best to propose them.

**Table 1.** $E_{\mathrm{reduction}}^{\circ }$ of all compounds in this study calculated at B3LYP/6-311++G** and SMD solvation model. For complete half-reactions in acid and base, refer to the electronic supplementary material (calculations.xlsx). The calculated difference in energy between H_3_O^+^ and H_2_O, ${\Delta G}_{\mathrm{soln}}^{*}=254.0\;\text{kcal}\;\text{mo}{\text{l}}^{-1}$ was used for H^+^. Calculated *E*^O^ for 2H^+^ + 2e^−^ ⇌ H_2_ is 4.98 V.

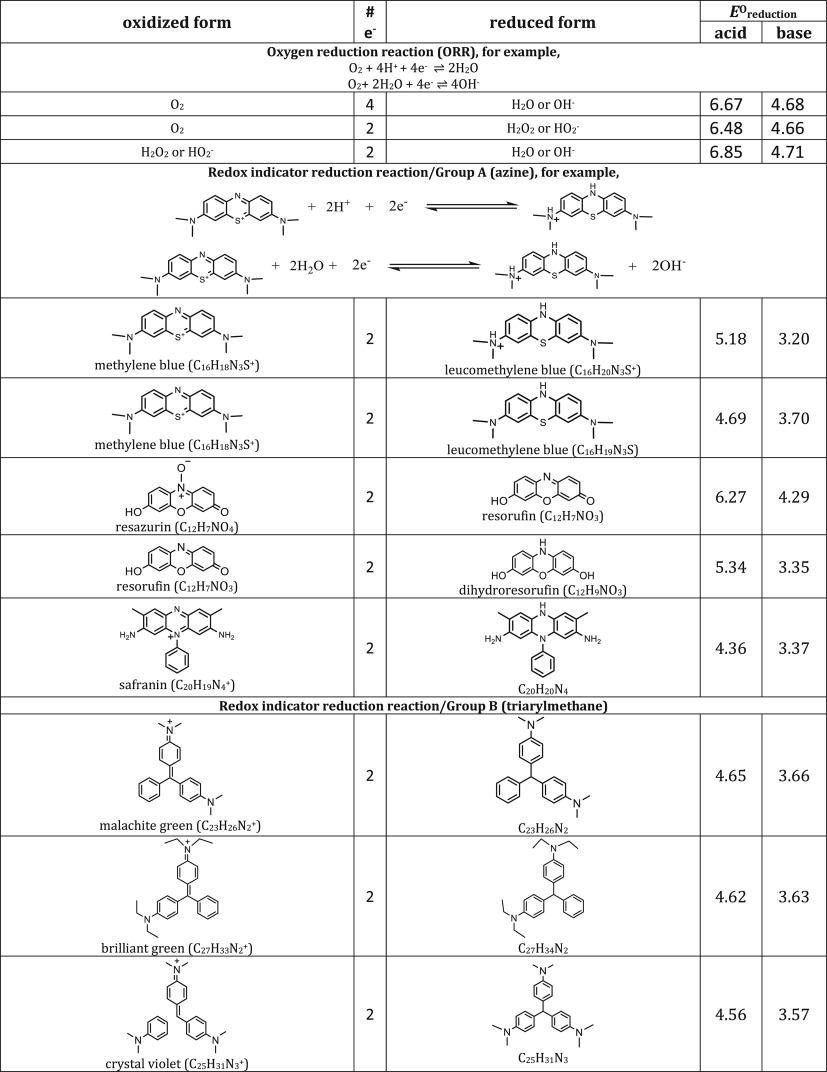
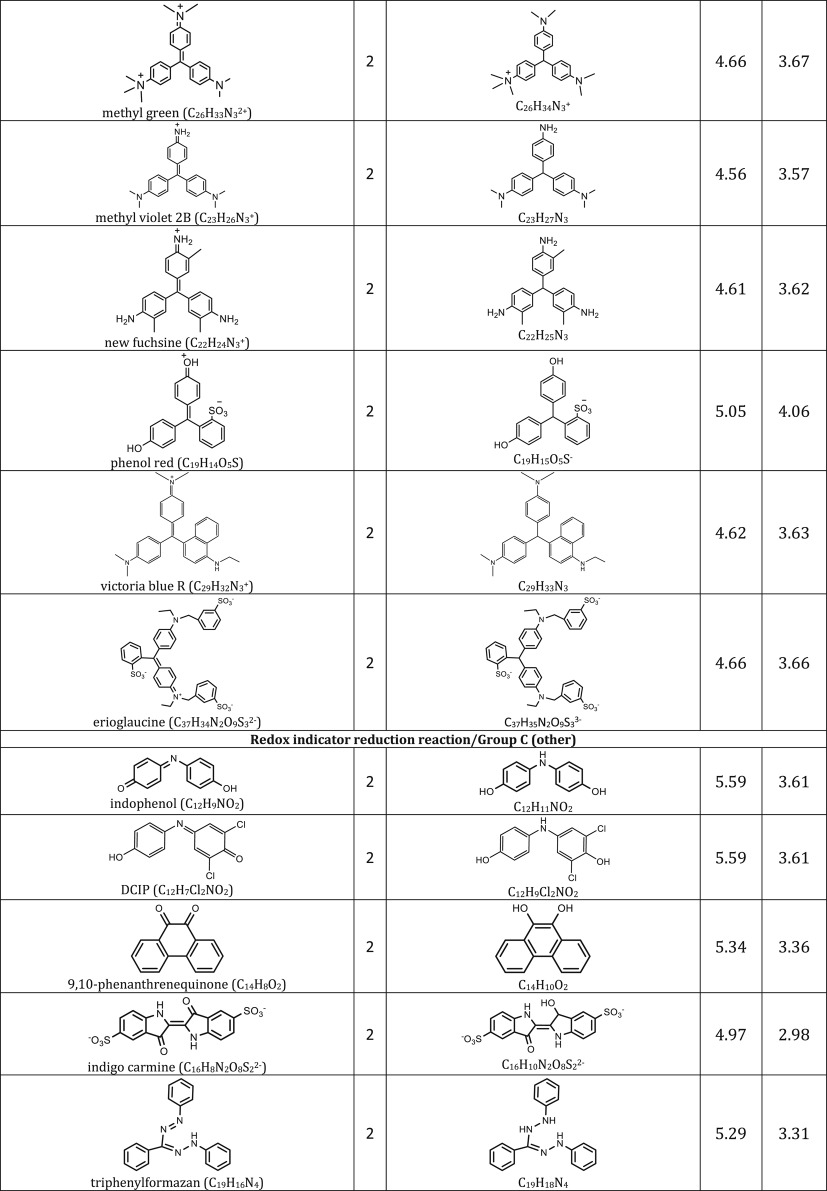
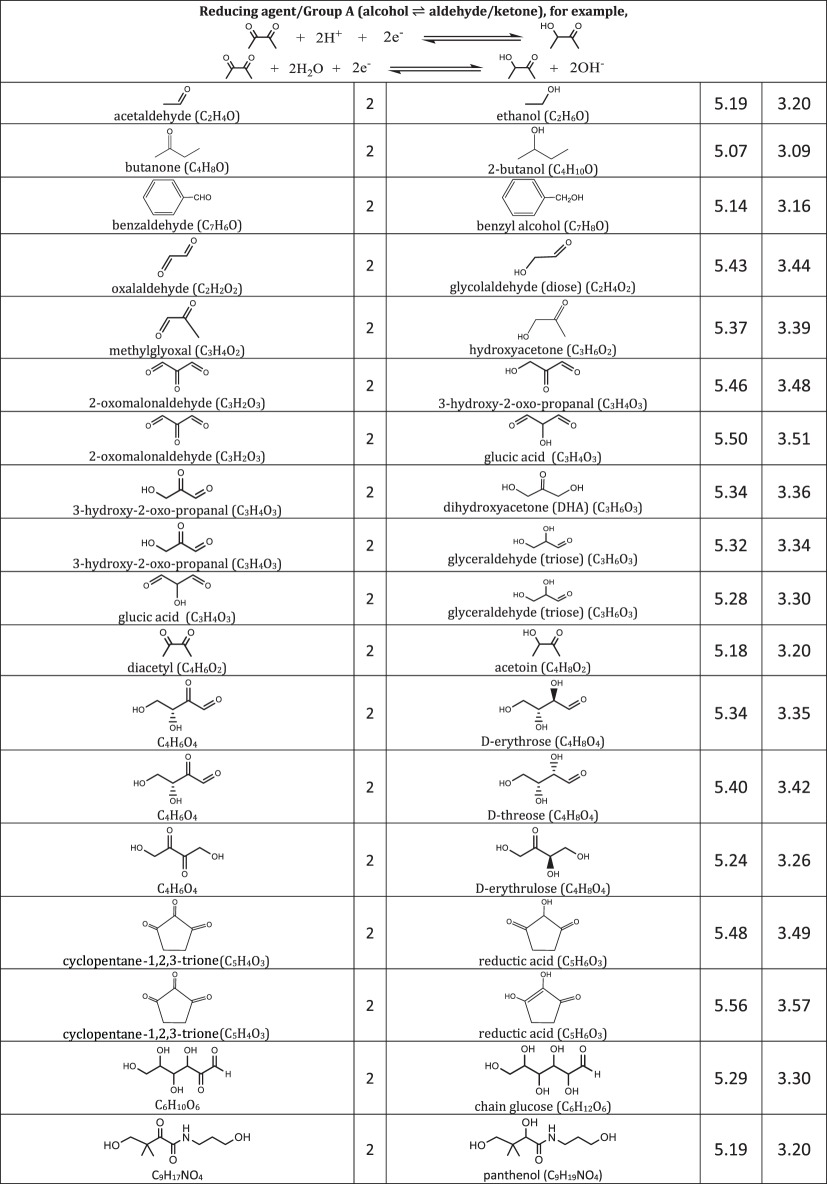
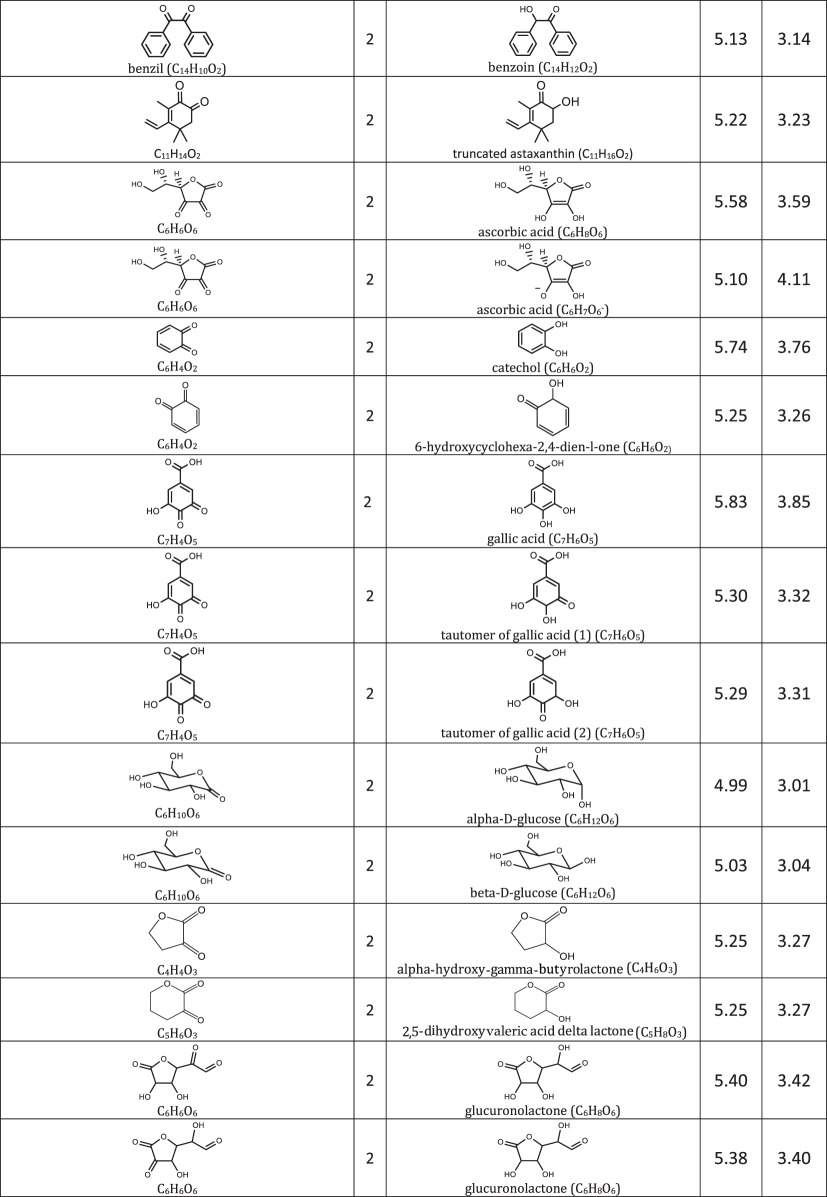
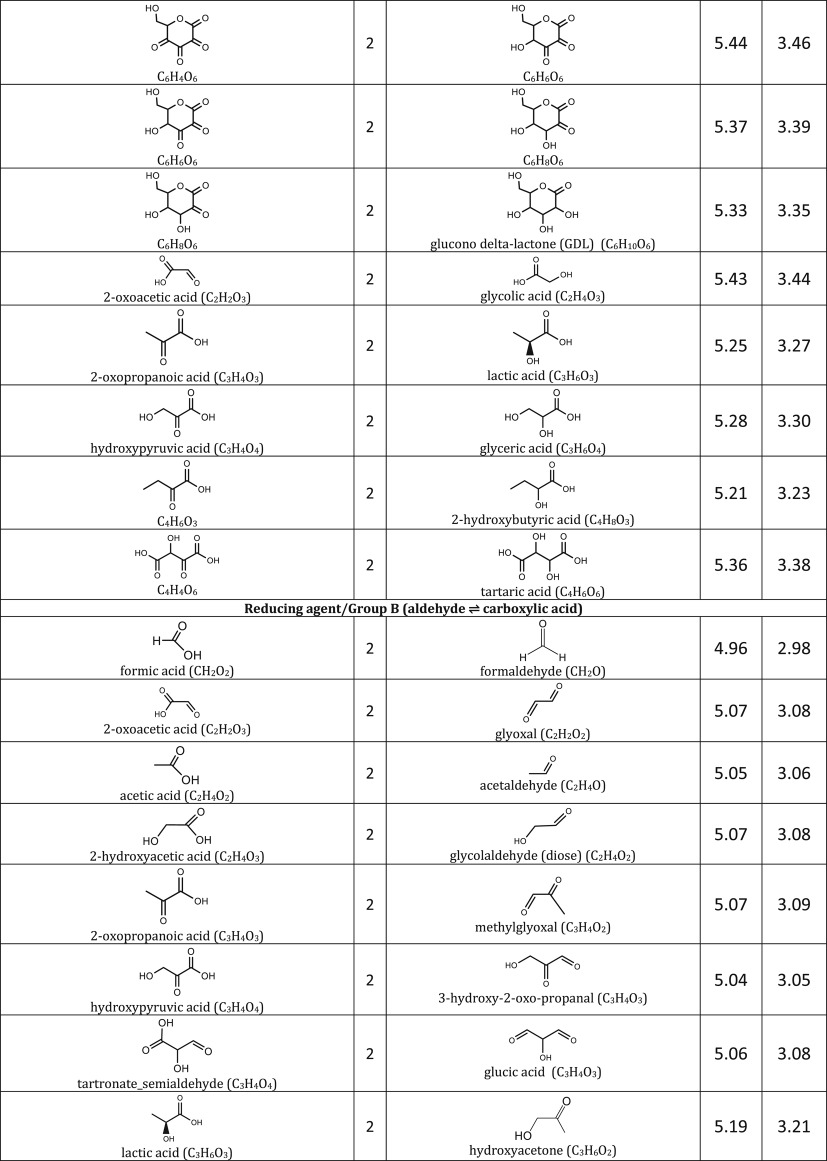
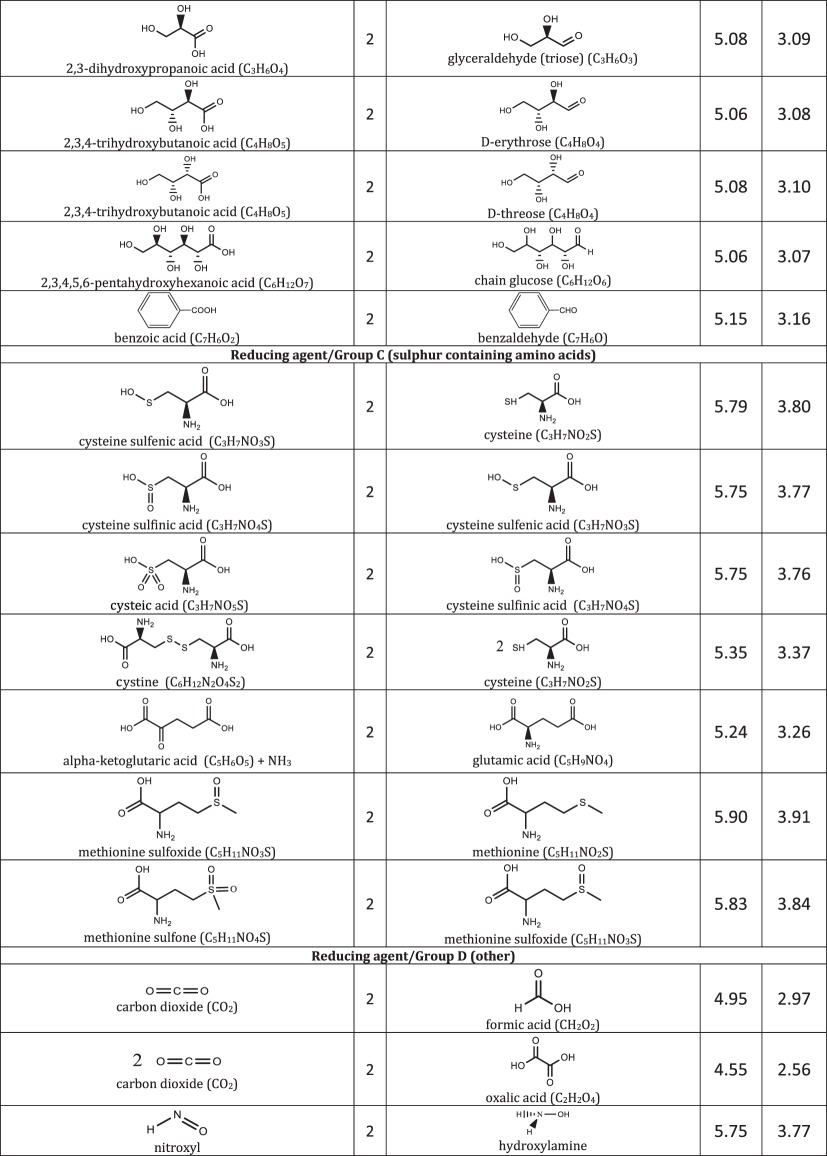
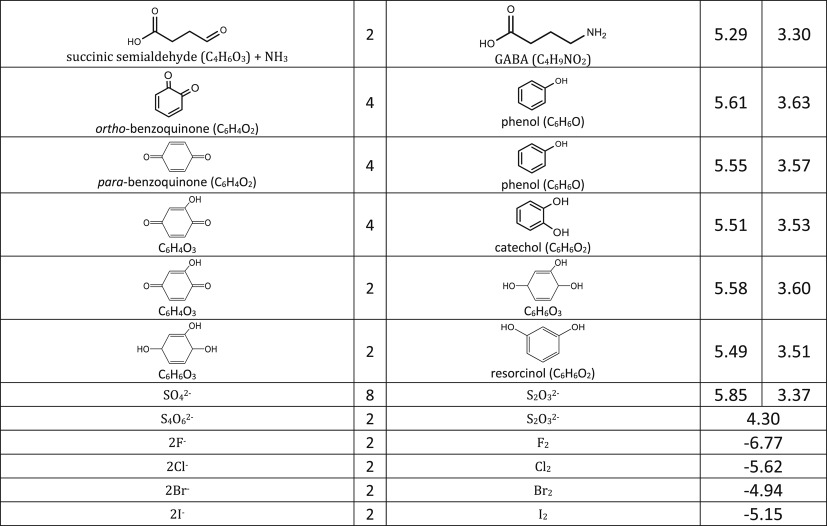

### 2.2. Grouping of oxygen reduction reactions, dyes and reducing agents

Reactions reported in the current literature and our proposal for dyes and reagents are studied as follows (structurally similar compounds are grouped together):

1. The ORRs are trivial but instead of using the literature values [30,31], the calculations were completed to obtain reference values for comparison purpose.
2. The dye oxidation/reduction reactions are grouped into:
  (i) heterocyclic azine: oxazine, thiazine and pyrazine,
  (ii) triarylmethane, and
  (iii) other common redox dyes.
3. The oxidation of reducing agents are grouped into
  (i) alcohol ⇌ aldehyde/ketone
    — simple substrates
    — acyloin including phenols, pyranoses and lactones
    — α-hydroxy carboxylic acids,
  (ii) aldehyde ⇌ carboxylic acid,
  (iii) sulfur-containing amino acids, and
  (iv) other common reducing agents.

### 2.3. Computational details

Different procedures [32–50] exist for computation of accurate $E_{\mathrm{half}\text{-}\mathrm{cell}}^{\circ }$ especially in one-electron case and/or families of structurally similar compounds [32–36,42,44–46,48]. Since all species in our study are simply closed-shell singlet organic molecules, however, a gas-phase DFT optimization followed by a free energy of solvation calculation has been proven successful in many cases [40,43,47–48]. This approach was also included and tested in recent reviews [49–51].

Gas-phase geometries were obtained at B3LYP/6-311++G** level and were confirmed to be a minimum point on the potential energy surface by frequency calculation. Solvation was treated by SMD model [52] on the gas-phase structure. Some compounds in our study have a number of rotamers and diastereomers. We try to use the lowest energy structure as a representative. However, the difference due to these stereoisomers is expected to be small (1 kcal mole^−1^ of electron is approximately 0.04 V). All output files are provided in the electronic supplementary material. (Additional calculation at B3LYP/6-31G* (gas phase) and MP2/cc-pVTZ (solution phase) were also completed on selected compounds for the preparation of initial structures for B3LYP/6-311++G** and for benchmarking, respectively.)

All calculations were performed using the Q-Chem 4.4 developer version [53]. The half-cell reduction potential was directly calculated from these equations:

For a half-cell reaction:

2.3 $$\Delta {G^{*}}_{\mathrm{soln}}=-nFE^{\circ }.$$

For a chemical structure [49]:

2.4 $$\begin{array}{rl} {G^{*}}_{\mathrm{soln}} & ={H^{\circ }}_{\mathrm{gas}}-T{S^{\circ }}_{\mathrm{gas}}+\;\Delta G_{\mathrm{solv}}, \end{array}$$

2.5 $$\begin{array}{rl} {H^{\circ }}_{\mathrm{gas}} & =\varepsilon_{0}(\text{gas})+H_{\mathrm{corr}}(\text{gas}), \end{array}$$

where $\Delta G^{\circ }$ is the standard Gibbs energy of the half reaction, *n* is the number of electrons in the half reaction, *F* is the Faraday constant 96 485 C mol^−1^, *G* is the standard Gibbs energy, *H* is the standard enthalpy, *T* is 298.15 K, *S* is the standard entropy, *ΔG*_solv_ is the free energy of solvation from SMD and standard state correction, *ε*_0_ is the electronic energy (*E*_B3LYP_ or *E*_MP2_ = *E*_HF_ + *E*_MP2 correlation_ as applicable) and *H*_corr_ is the total enthalpy correction to *ε*_0._

### 2.4. Standard state, reference potential and deviations

In solution phase, 1 M reference state is used with the exception of water where 55.34 M is used [54–58]. The correction for these are 3.02 mhartree and 3.80 mhartree, respectively. We do not use reference potential and ignore electrons in $\Delta G^{\circ }$ calculation.

Most variations of the blue bottle experiments take place in a alkaline solution with an exception of ascorbic acid system. The reactions are therefore considered in acid and alkaline conditions separately. Hydronium ion ([H_3_O^+^] = 1 M, pH = 0) and water are used in the calculation instead of hydrogen ion (H^+^) for reactions under acidic condition. Similarly, hydroxide ion ([OH^−^] = 1 M, pH = 14) is used for reactions under alkaline condition. Examples are provided in table 1 to illustrate reactions under acidic and alkaline conditions.

Reduction potentials of many redox reactions are pH dependent due to deviation from standard condition. The deviation in terms of Gibbs energy (*RT* ln *Q*) is expressed in the last term of the Nernst equation,

2.6 $$E=E^{\text{O}}-\frac{RT}{nF}\ln Q,$$

where *Q* is the reaction quotient. Since pH is not exactly 0 or 14 and the concentration of reactants are generally lower than 1 M, the ln *Q* consideration may be employed for detail analysis, especially when *E*_cell_ is close to zero.

### 2.5. Benchmarking

**Figure 3.**
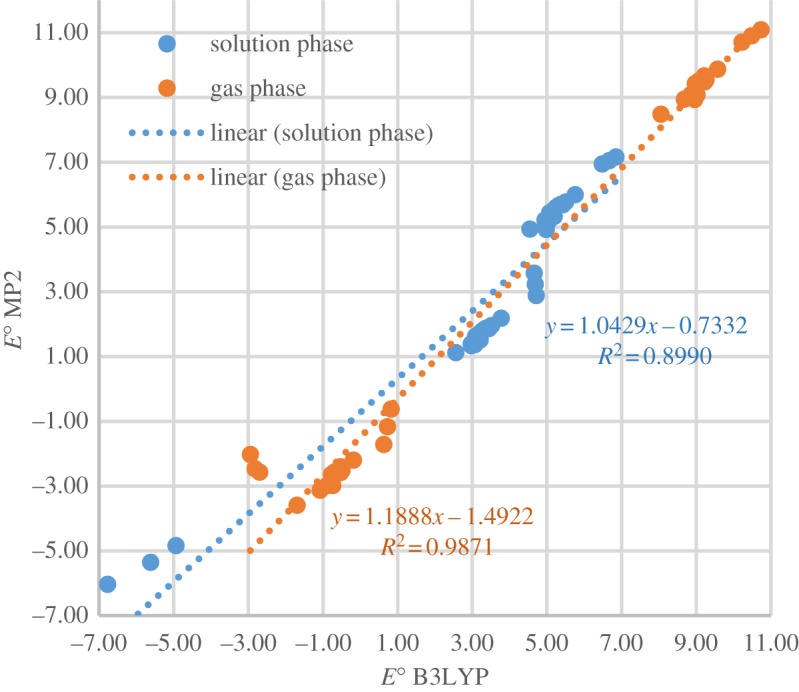
Comparison of reduction potentials of reactions at B3LYP/6-311++G** and MP2/cc-pVTZ levels.

The mean unsigned errors for solution-phase and gas-phase *E*^O^ of 52 selected reactions obtained at B3LYP/6-311++G** and MP2/cc-pVTZ are 0.86 V and 1.10 V, respectively. Figure 3 shows satisfactory linear relationships between *E*^O^ obtained by the two methods (high *R*^2^ value but slope values slightly greater than unity). These benchmarking results confirm that B3LYP/6-311++G** yields acceptable results at a relatively small computational cost [59].

## 3. Results and discussion

### 3.1. Reduction potentials

**Figure 4.**
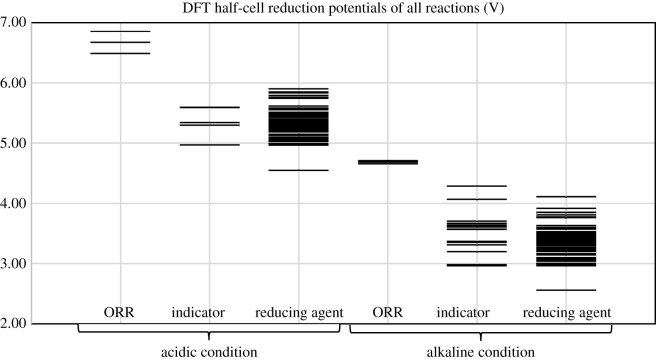
Reduction potentials of reactions in acidic and alkaline conditions calculated at B3LYP/6-311++G** level.

Table 1 shows half-cell reduction potentials of all possible reactions in the blue bottle experiment. For comparison purpose, figure 4 shows the reduction potentials in acidic and alkaline conditions for the three groups of compounds, respectively. The first set of data is four- and two-electron oxygen reduction potentials on the first and fourth column of figure 4. The values are far from the literature values [30,31] but the trend that acidic potentials are higher than alkaline potentials is still preserved. The second set of data is the dye reduction potentials. Figure 4 shows that all dyes including indophenol may be conveniently oxidized by either two- or four-election ORR. The third set of data is the reducing agent reduction potentials. (Refer to Methodology section for grouping of reactions. Some compounds, for example, glucose, belong to two groups of reactions.)

In general, the reduction potentials are quite similar for reactions in the same group under the same condition, and acidic potentials are higher than alkaline potentials. As reduction potentials of dyes and reducing agents are overlapping in figure 4, there are combinations of dyes and reducing agent that may or may not work in the blue bottle experiment.

### 3.2. Thermodynamic considerations

Positive cell potential from combination of the compounds can be found in figure 4 if the half-cell potentials decrease from left to right. For example, in alkaline condition, the classical blue bottle experiment may proceed via two- or four-electron oxygen reduction (approx. 4.7 V), with methylene blue as a catalyst (3.70 V) and glucose as a reducing agent (3.30 V). In acidic condition, the green version of the blue bottle experiment may proceed via two- or four-electron oxygen reduction (6.5–6.8 V), with methylene blue as a catalyst (5.18 V) and ascorbic acid as a reducing agent (5.10 V).

As our reduction potential considerations here are thermodynamic, the negative prediction (non-spontaneity for large negative value of $E_{\mathrm{cell}}^{\circ }$) should be valid but the positive prediction (combination of dye and reducing agent make a blue bottle reaction for positive or close to zero value of $E_{\mathrm{cell}}^{\circ }$) requires further verifications. To produce repeatable cycle of colour change, the rate of reduction of dye by reducing agent must be slower than the oxidation of dye by oxygen [14] and the direct oxidation of reducing agent by oxygen [5] should be minimal compared with the dye-catalysed reaction. Additional catalysts similar to the green version of the experiment [8,16] may be needed to make the reaction occur but it is beyond the scope of this study.

### 3.3. Implications and experimental confirmation

The following findings are made based on the calculated results and information in the literature. To support our claims, preliminary experiments to test some reducing agents were also carried out (see the electronic supplementary material).


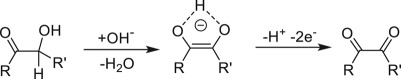

1. Acyloin/enediol structure is necessary for the blue bottle reaction [5,7,8,11,16] and the main product is 1,2-diketone.
  — Replacement of dextrose by simple aldehydes, e.g. benzldehyde, and alcohols, e.g. ethanol, does not result in repeatable colour change. The solution is blue and is not reduced to colourless over time.
  — Replacement of dextrose by acetoin and dihydroxyacetone in the blue bottle experiment yields repeatable colour change and chemical patterns [18]. Phenol-derivatives such as catechol reacts with oxygen rapidly under alkaline conditions without a dye to produce a dark-coloured solution.
  — GC/MS analysis of rapid blue bottle experiment confirms that benzil is a product of the reaction.
2. The mechanism of reduction proceeds via enediolate formation [5] (deprotonation at carbon attached to OH).
  — It is possible for ascorbic acid to deprotonate without the use of base and it is the only reducing agent for the experiment in acidic condition.
  — Replacement of dextrose by *α*-hydroxy carboxylic acids such as citric acid and tartaric acid does not result in repeatable colour change. The solution is blue and it is not reduced to colourless over time. For citric acid, the tertiary alcohol group cannot be oxidized to ketone. For, tartaric acid, the negative charge upon deprotonation of –COOH group may make it difficult for the oxidation of the adjacent hydroxyl group.
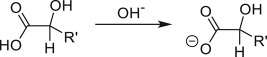
  — Replacement of dextrose by α-hydroxy lactones, for example, glucono delta-lactone did not change the colour of the dye from blue to colourless.
3. Alternative reducing agents such as amino acids may also be used in the blue bottle experiment.
  — Replacement of dextrose by amino acid and various food products yields repeatable colour change but some of the reactions are slow [18].
  — In an iodine clock experiment [60], cysteine is also used successfully in place of ascorbic acid.

## 4. Concluding remarks and future work

Half-cell reduction potentials of oxygen, redox indicators and reducing agents have been investigated using DFT calculations. The results help us better understand the blue bottle reaction and guide us to focus the experiments only on a certain number of representative compounds and only for reaction that lead to a positive cell potential. The use of alternative reducing agents can help avoid side reactions that make the solution brown after a number of cycles [5] and increase solubility of the reducing agent in water which is a known issue for benzoin [7]. Possible future computational investigation includes prediction of p*K*a [39,56] and stability of intermediates and activated complexes of reduction reactions and prediction of the colour of redox dyes [61].

## Supplementary Material

calculations.xlsx

## Supplementary Material

output.zip

## Supplementary Material

experiment.pdf

## Supplementary Material

omp2.txt

## Supplementary Material

ob3lyp.txt

## Data accessibility

The data supporting the findings of this study are available in the electronic supplementary material.

— Energies and graphs are summarized in calculations.xlsx.
— Preliminary experimental results are in experiment.pdf.
— Optimized geometries are in ob3lyp.txt and omp2.txt.
— All Q-Chem output files are in output.zip
