# Correction to ‘A DFT investigation of the blue bottle experiment: *E*^○^_half-cell_ analysis of autoxidation catalysed by redox indicators’

**DOI:** 10.1098/rsos.171563

**Published:** 2017-11-08

**Authors:** Taweetham Limpanuparb, Pakpong Roongruangsree, Cherprang Areekul

*R. Soc. open sci.* **4**, 170708. (Published Online 1 November 2017). (doi:10.1098/rsos.170708)

**List of changes and reasons:**

Table 1: errors and omissions for some entries of the table

— Half-cell potentials for butanone and halogens were corrected.
— Additional structures (indigo carmine derivatives and reducing agents for iodine clock reaction) and their half-cell potentials were added.

Figures 3 and 4 and numerical values in Benchmarking section were updated to reflect the change in table 1.

The corrected version of table 1, figures 3 and 4 and text in benchmarking section are shown below:

**Figure 3.**
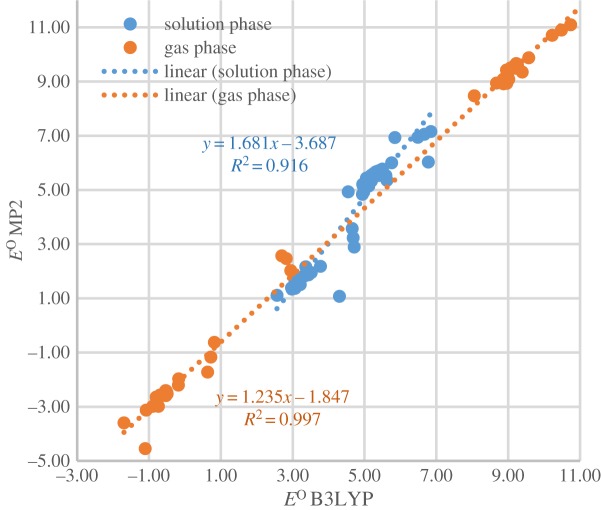
Comparison of reduction potentials of reactions at B3LYP/6-311++G** and MP2/cc-pVTZ levels.

**Figure 4.**
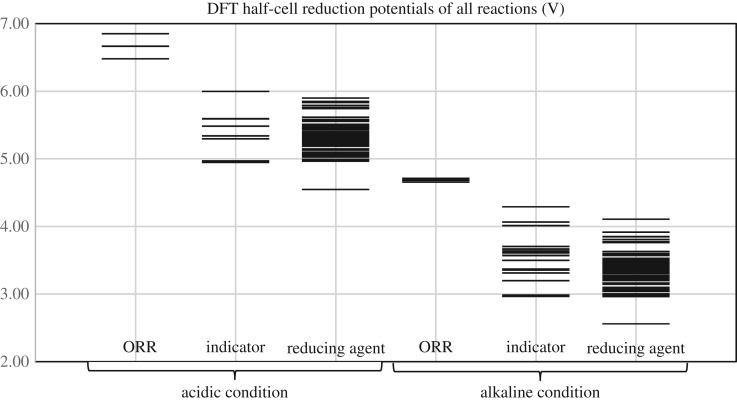
Reduction potentials of reactions in acidic and alkaline conditions calculated at B3LYP/6-311++G** level.

**Table 1.** $E_{\mathrm{reduction}}^{\circ }$ of all compounds in this study calculated at B3LYP/6-311++G** and SMD solvation model. For complete half-reactions in acid and base, refer to the electronic supplementary material (calculations.xlsx). The calculated difference in energy between H_3_O^+^ and H_2_O, $\Delta {G^{*}}_{\mathrm{soln}}=254.0\;\text{kcal}\;\text{mo}{\text{l}}^{-1}$ was used for H^+^. Calculated *E*^O^ for 2H^+^ + 2e^−^ ⇌ H_2_ is 4.98 V.

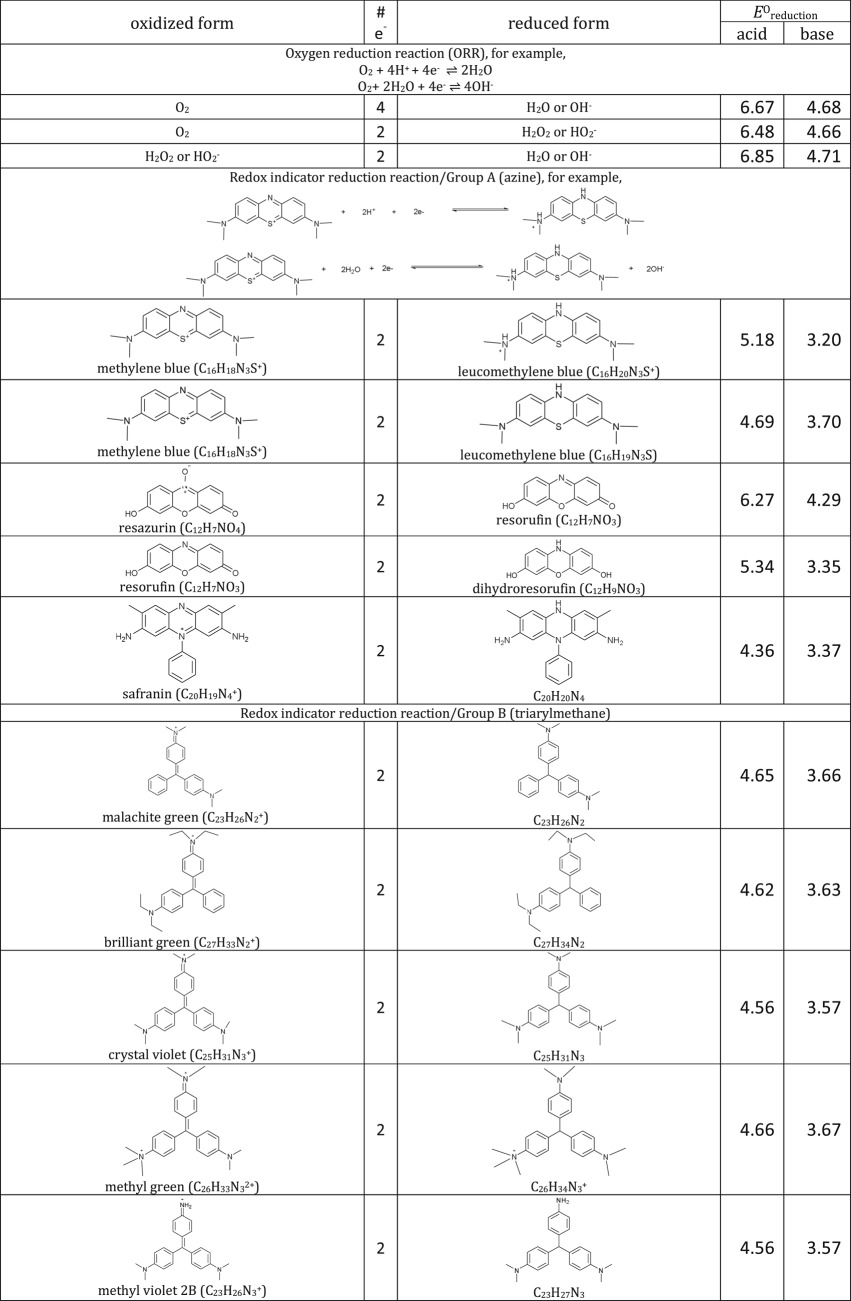
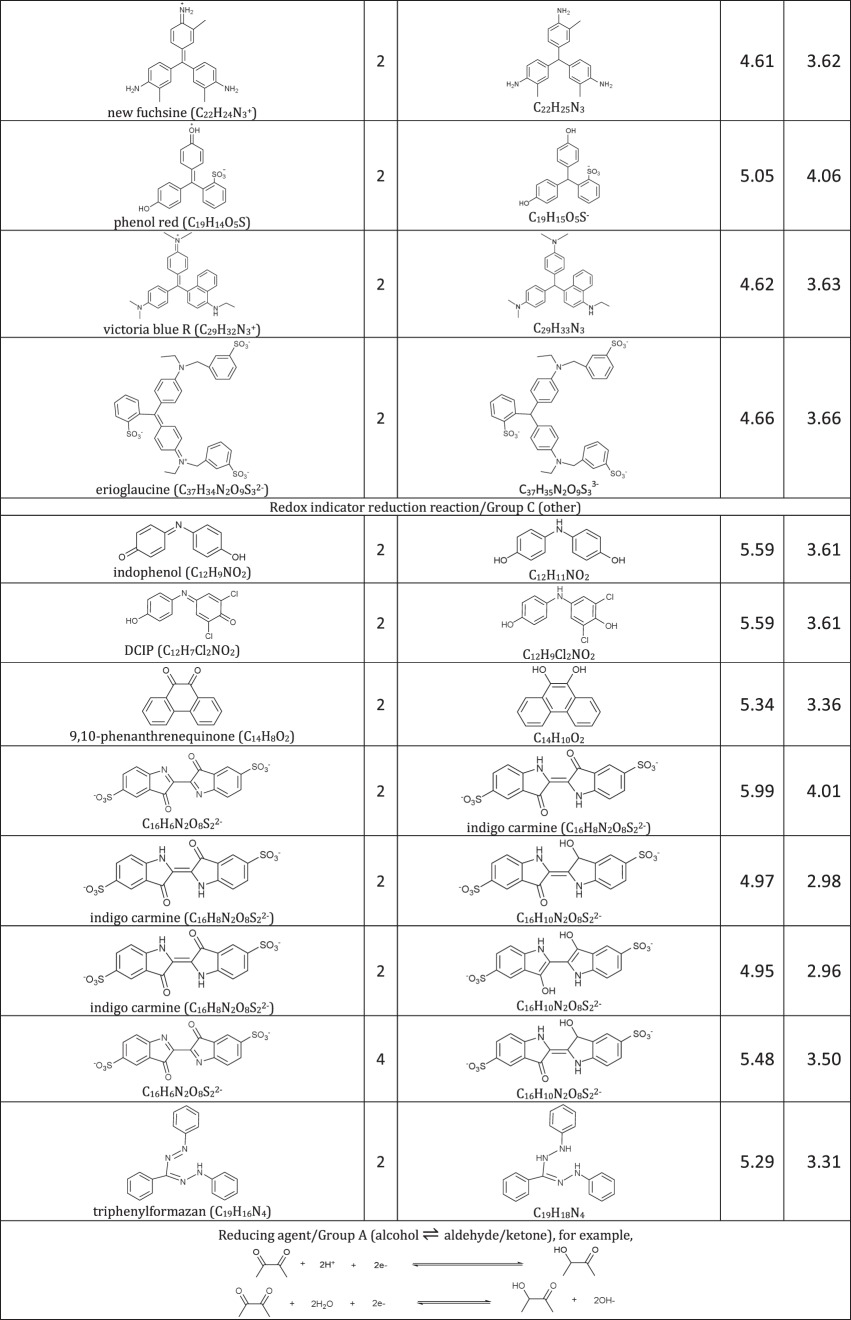
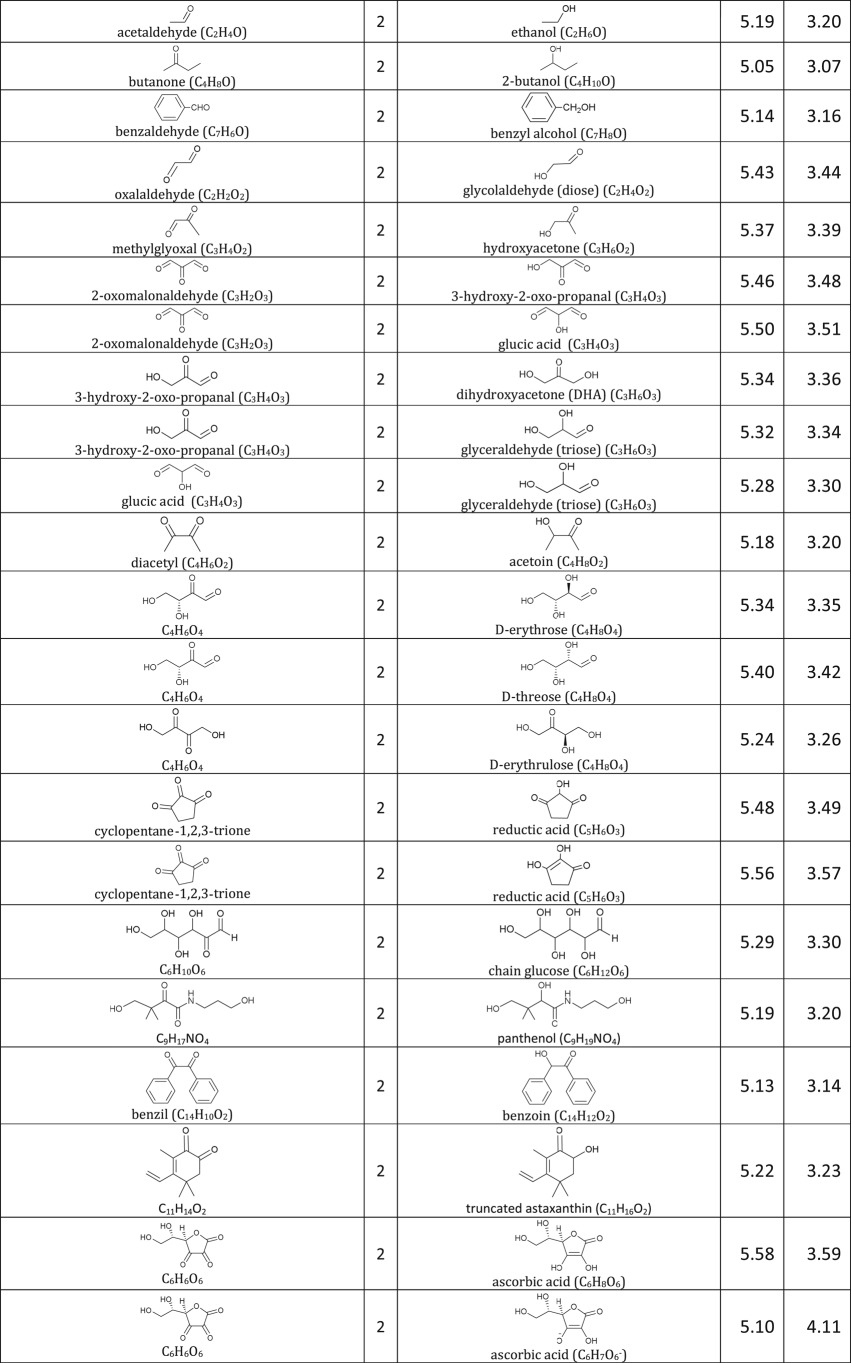
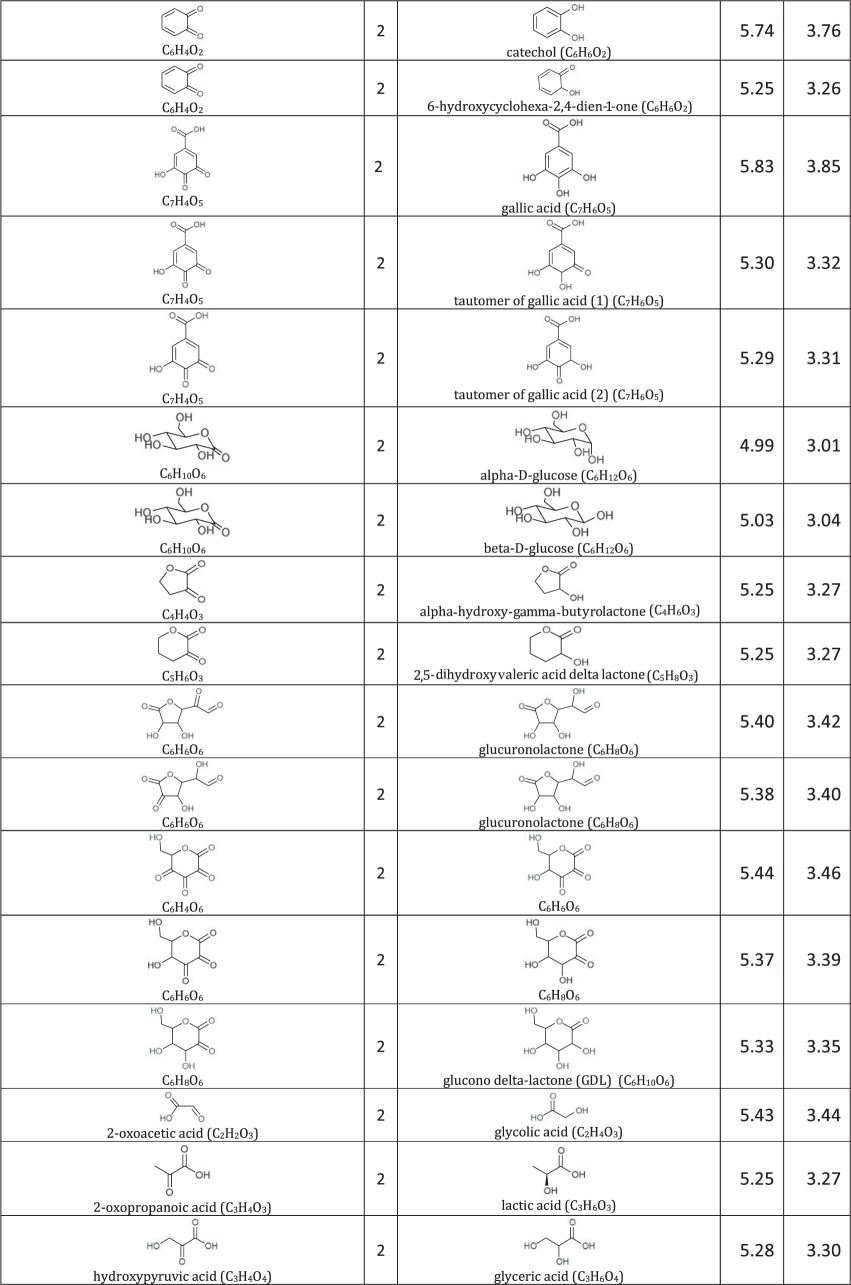
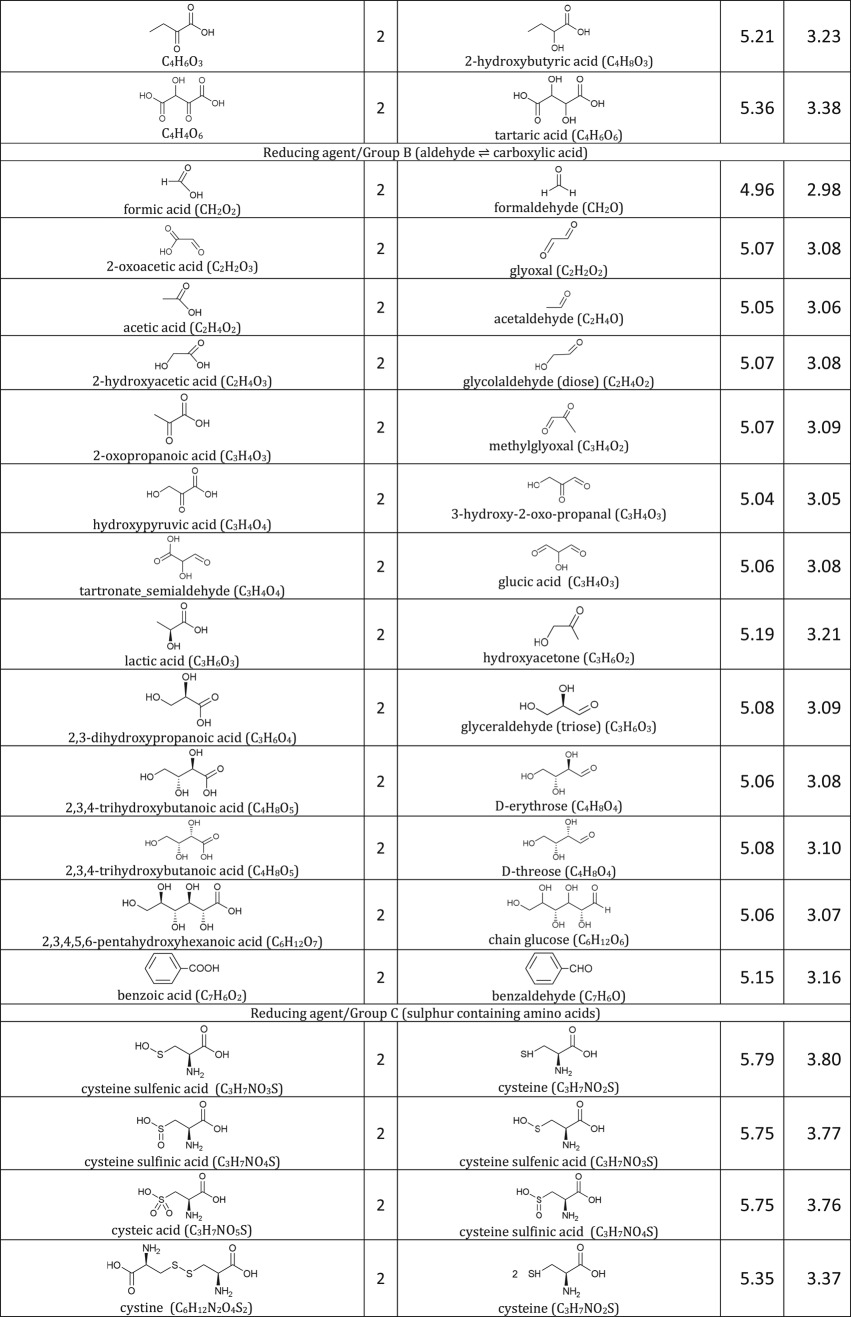
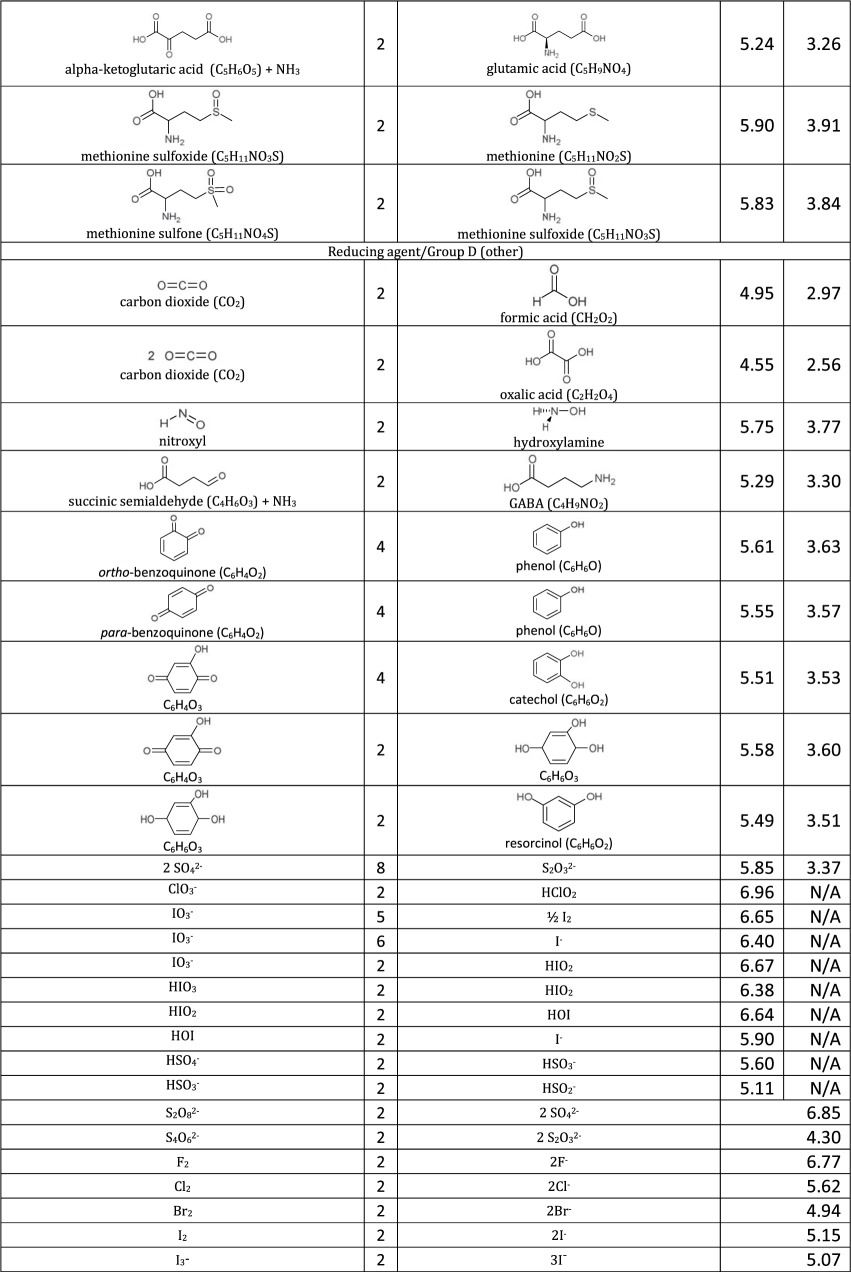

## Benchmarking

The mean unsigned errors for solution-phase and gas-phase *E*^O^ of 57 selected reactions obtained at B3LYP/6-311++G** and MP2/cc-pVTZ are 0.89 V and 1.11 V, respectively. Figure 3 shows satisfactory linear relationships between *E*^O^ obtained by the two methods (*R*^2^ values are high but slope values greater than unity). These benchmarking results confirm that B3LYP/6-311++G** yields acceptable results at a relatively small computational cost [59].

## Supplementary Material

Detailed worksheet for energies and graphs

## Supplementary Material

All Q-Chem output files and optimised geometries

## Data accessibility

The data supporting the findings of this study are available in the electronic supplementary material.

— Energies and graphs are summarized in calculations.xlsx.
— All Q-Chem output files and optimized geometries are in output.zip.

