# Correction to ‘A DFT investigation of the blue bottle experiment: *E°*_half-cell_ analysis of autoxidation catalysed by redox indicators’

**DOI:** 10.1098/rsos.200115

**Published:** 2020-02-05

**Authors:** Taweetham Limpanuparb, Pakpong Roongruangsree, Cherprang Areekul

*R. Soc. open sci.* **4**, 171563. (Published 8 November 2017). (doi:10.1098/rsos.171563)

This correction refers to a misprint of the sign (±) in figure 1. The original paper has now been corrected.

