# Supplementary material for: A DFT investigation of the blue bottle experiment: E∘half-cell analysis of autoxidation catalysed by redox indicators
Source: R Soc Open Sci. 2017 Nov 8;4(11):170708. doi: 10.1098/rsos.170708 (PMC5717635; doi:10.1098/rsos.170708)

Preliminary experimental evidence

1. Benzil GS/MS confirmation
2. The blue bottle experiment based on alternative reducing agents

Benzil GS/MS confirmation

```
File        :D:\GCMS_Data\Pakorn\2016_May\18_May_2016\TWL_BENZOIN.D
Operator    :
Acquired    : 18 May 2016   15:45      using AcqMethod AGILENTNEW300.M
Instrument  :    GCMSD
Sample Name: TWL_BENZOIN
Misc Info   :
Vial Number: 10
```

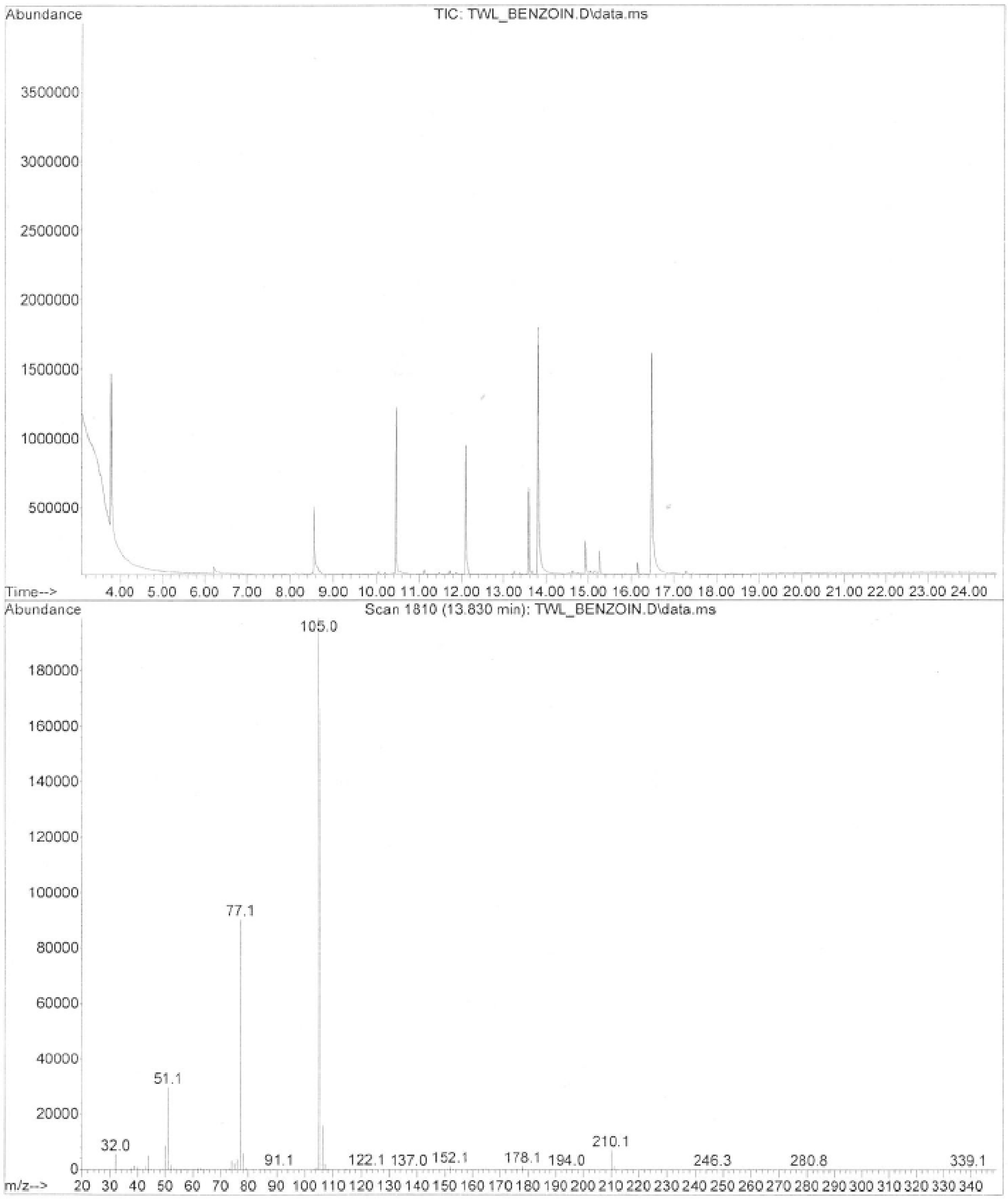

```
File        :D:\GCMS_Data\Pakorn\2016_May\18_May_2016\TWL_BENZOIN.D
Operator    :
Acquired    : 18 May 2016  15:45     using AcqMethod AGILENTNEW300.M
Instrument :    GCMSD
Sample Name: TWL_BENZOIN
Misc Info   :
Vial Number: 10
```

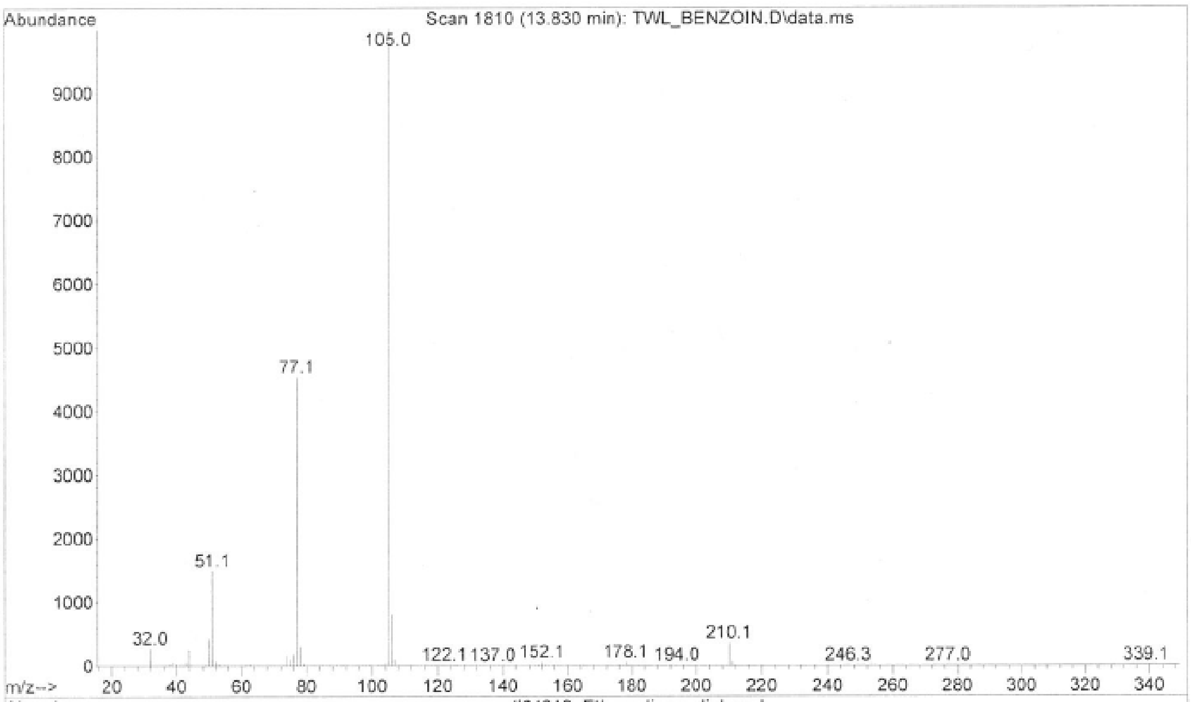

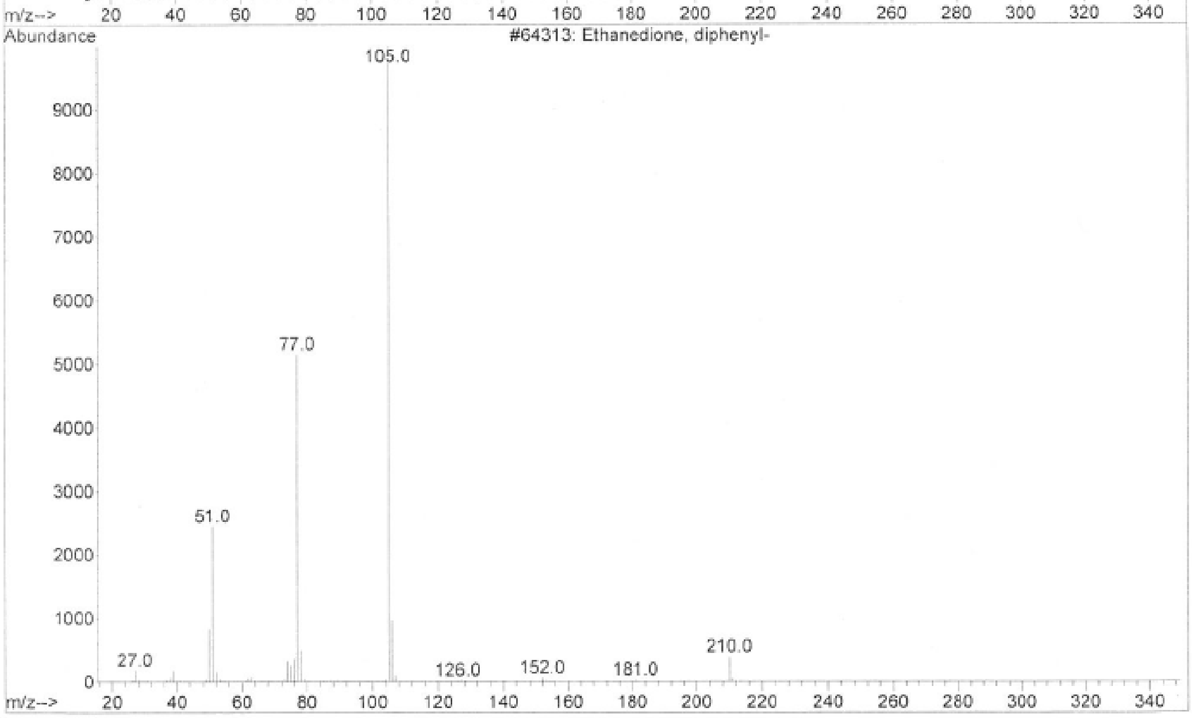

The blue bottle experiment based on alternative reducing agents

Acetoin

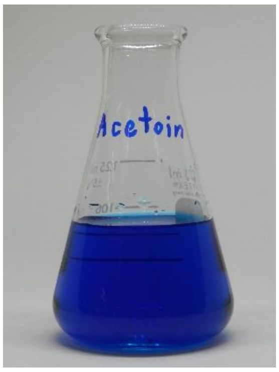

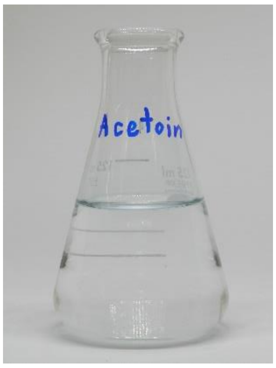

Cysteine

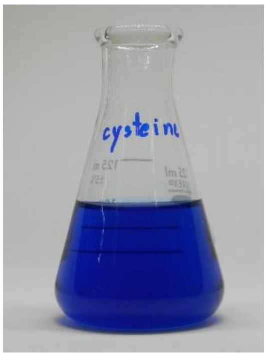

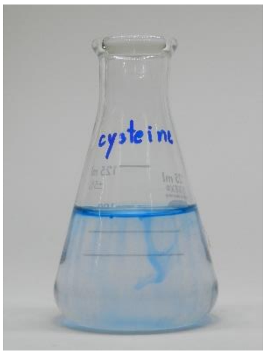

Supplement: experiment.pdf [file rsos170708supp3.pdf]
